# Prediction and visual analysis of flue-cured tobacco aroma types based on machine learning and feature derivation

**DOI:** 10.1039/d6ra00543h

**Published:** 2026-05-20

**Authors:** Zhiying Wang, Shouchen Yang, Xiaoting Wang, Jun Qiu, Jianmin Cao, Xianwei Hao

**Affiliations:** a Tobacco Research Institute of CAAS Qingdao 266101 China 821012450666@caas.cn caojianmin@caas.cn +86-532-88703386 +86-18375487707 +86-13864806225; b Graduate School of CAAS Beijing 100081 China; c Technology Center, China Tobacco Shandong Industry Co., Ltd Qingdao 266101 China; d Technology Center, China Tobacco Zhejiang Industrial Co. Ltd Hangzhou 310000 China hxwzjzy2020@163.com +86-571-81188181 +86-15968175953

## Abstract

The present study aimed to investigate the relationship between aroma types and chemical properties of flue-cured tobacco (FCT), and to explore the applicability of machine learning (ML) combined with feature derivation in the FCT industry. A total of 619 Sichuan FCT samples representing three aroma types (fresh-sweet, honey-sweet, and mellow-sweet) were utilized. Feature derivation was performed based on 51 raw chemical indices, followed by a three-tier key indicator selection process incorporating separability analysis, Random Forest (RF) importance ranking, and redundant feature elimination *via* correlation analysis. By comparing multiple machine learning models, the optimal model adapted to the Sichuan FCT dataset was screened out. Model parameter optimization was accomplished in combination with the genetic algorithm (GA), and finally, visual interpretation of the model's decision-making mechanism was realized by means of SHAP values. The results demonstrated that after three-tier screening, 9 key characteristic indices including rutin-malonic acid, rutin and chlorogenic acid et al were finally identified. The random forest (RF) algorithm was the optimal model for this dataset; after parameter optimization, the model achieved an *F*1-score of 88.3% and an accuracy of 93.5%, which greatly reduced the detection cost and improved the model's discrimination performance. Additionally, the SHAP value interpretation framework clearly reveals the intrinsic correlation between chemical characteristics and aroma types. This study not only enhances the efficiency of aroma type classification for Sichuan FCT but also clarifies the key chemical indicators associated with aroma traits. It further provides quantitative support for optimizing FCT quality through the targeted regulation of key component contents.

## Abstract 2

The present study aimed to investigate the relationship between aroma types and chemical properties of flue-cured tobacco (FCT), and to explore the applicability of machine learning (ML) combined with feature derivation in the FCT industry.

## Graphical abstract

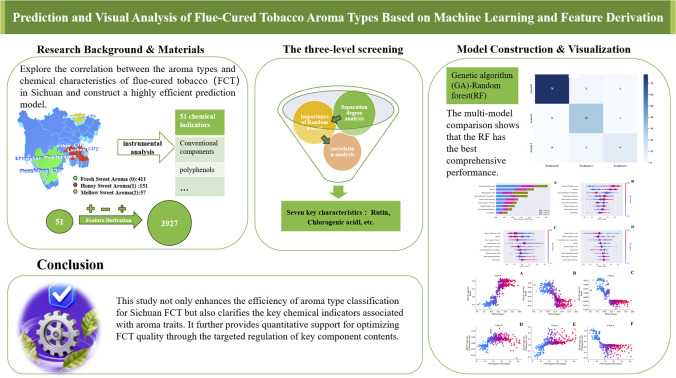

## 1 Introduction

Tobacco is a globally important economic crop, widely cultivated in China, the United States, Brazil, and other countries.^1^ Under the influence of different geographical environments, climatic conditions, and agricultural management practices, the same tobacco variety exhibits significant differences in chemical properties and aroma characteristics, which in turn lead to obvious variations in tobacco aroma style and quality.^2,3^ In China, a classification standard for eight major aroma types of FCT has been established through multi-dimensional classification and cross-validation based on ecological conditions, aroma characteristics, chemical compositions, and metabolic mechanisms.^4^

To clarify the intrinsic correlation between tobacco aroma types and chemical compositions, researchers have conducted extensive studies in recent years by combining multivariate statistical analysis with modern instrumental analysis techniques.^5–7^ For example, some studies have performed qualitative and quantitative evaluation of the smoke aroma quality and five categories of flavor-related chemicals of honey-sweet and burnt aroma types of Shandong FCT through a combination of sensory analysis and instrumental analysis, and identified the key chemical components determining its honey-sweet and burnt-sweet aroma characteristics.^8^ Based on molecular sensory science, other studies have analyzed the unique honey-sweet flavor of Guizhou FCT by combining the analysis of volatile components, cell wall components, and odor activity value (OAV) assessment, confirming that 8 core aroma-active substances are the key markers distinguishing it from FCT from other producing areas. However, this study only focused on the aroma components of the single honey-sweet type of Guizhou FCT and did not involve relevant research on aroma type identification.^9^ In addition, a study has established a rapid quantitative analysis model for aroma components using gas chromatography-tandem mass spectrometry (GC-MS/MS) and combined it with chemometric methods to achieve accurate identification of three FCT aroma types, namely fresh-sweet aroma, honey-sweet, and burnt-sweet mellow.^10^

Against this background, machine learning (ML) algorithms have developed rapidly in various fields, and their applications in the tobacco industry have also been actively promoted.^11–13^ As a popular branch of artificial intelligence, ML algorithms are proficient in processing multi-dimensional data, capable of effectively removing redundant information, extracting intrinsic laws, revealing complex relationships between dimensions, and are free from subjective interference throughout the process, thereby satisfying the requirements for in-depth mining of multi-dimensional tobacco chemical data.^14^ However, most relevant studies in the tobacco field currently only focus on the direct correlation between raw chemical indicators and tobacco quality,^15^ and research on conducting feature derivation based on raw indicators followed by key feature screening and modeling is still relatively scarce. This shortcoming not only limits the in-depth analysis of the intrinsic mechanism between tobacco aroma types and chemical components, but also restricts the efficiency and model accuracy of tobacco aroma type discrimination.

Sichuan is a major producer of high-quality tobacco leaves in China, with its output ranking among the top five nationwide, and serves as a core source of raw materials for Chinese-style cigarettes.^16^ Benefiting from a unique ecological environment, this region produces three different aroma types of FCT: fresh-sweet aroma, honey-sweet aroma, and mellow-sweet aroma.^4,16^ However, most relevant studies in the tobacco field only focused on the direct correlation between raw chemical indicators and tobacco quality,^17,18^ and research on conducting feature derivation based on raw indicators followed by key feature screening and modeling remains relatively scarce. To clarify the aroma-chemical correlation of Sichuan FCT, this study took Sichuan FCT samples as the research objects, aiming to deepen the understanding of the intrinsic relationship between aroma style and chemical substances, and improve the efficiency of aroma type discrimination. Specifically, the study first performed feature derivation based on 51 original chemical indicators, and then gradually screened key features through separability analysis, random forest (RF) importance ranking, and correlation analysis to balance the reduction of detection costs and the guarantee of model accuracy. Subsequently, the predictive performance of six ML algorithms—random forest (RF), *K*-nearest neighbor (*K*NN), decision tree (DT), logistic regression (LR), extreme gradient boosting (XGBoost) and partial least squares-discriminant analysis (PLS-DA)—was compared. Meanwhile, the SHAP value interpretation framework was introduced to clarify the impact of their value ranges on discrimination results. This study can enhance the interpretability of predictive data and provide new ideas and data support for the application of ML algorithms in the tobacco field and the evaluation of tobacco quality and style.

## 2 Materials and methods

### 2.1 Tobacco samples

In this experiment, FCT leaves from 5 representative producing areas (Liangshan, Panzhihua, Yibin, Luzhou, and Guangyuan) in Sichuan Province during the 2021–2023 growing seasons were selected. The sampling quantity was determined based on the planting area of each producing area, and a total of 619 samples of local main cultivars (including Yunyan 87 and Honghuadajinyuan) were collected. Aroma type evaluation of the experimental samples was performed in accordance with YC/T 530, a tobacco industry standard of the People's Republic of China. Based on the evaluation results, three aroma types were identified and assigned numerical values of 0, 1, and 2 respectively, to facilitate subsequent experimental work. Detailed information on the samples is provided in Table 1.

**Table 1:** Information table of samples

Aroma type	Representative regions	Sampling points/number	Numbers of samples
Fresh-sweet aroma (0)	Liangshan	47	411
	Panzhihua	15	
Honey-sweet aroma (1)	Yibin	12	151
	Luzhou	10	
Mellow-sweet aroma (2)	Guangyuan	10	57

### 2.2 Determination methods of chemical composition

Water-soluble sugars, total alkaloids, total nitrogen, potassium, chlorine, starch, protein, polyphenols, individual alkaloids, as well as polybasic acids and higher fatty acids were determined in accordance with the relevant tobacco industry standard of the People's Republic of China (YC/T standards). Free amino acid contents in samples were assayed following the method described by Zhang *et al.*^19^ while sugar alcohols were determined based on the protocol reported by Ghfar *et al.*^20^

### 2.3 Data processing methods

#### 2.3.1 Feature derivation

The chemical components determined in Section 2.2 were adopted as the original dataset for feature derivation. Given that the chemical properties of FCT are all continuous variables, addition, subtraction, and division operations were performed on the original features in this study to ensure the interpretability of inter-variable relationships. Using 51 indicators, including conventional chemical components, alkaloids, polyphenols, sugar alcohols, and amino acids, as raw features, 1326 additive, 1275 subtractive, and 1275 divisive composite features were generated *via* feature derivation. Together with the original 51 indicators, a dataset encompassing 3927 features was constructed (partially shown in Table 2).

**Table 2:** Feature-derived dataset (part)

Feature	Type	Feature	Type	Feature	Type
Total sugars	Primitive features	Total sugars + protein	Addition feature	Proline − sucrose	Subtraction feature
Reducing sugar	Primitive features	Total sugars + chlorine	Addition feature	Starch − potassium	Subtraction feature
Total alkaloids	Primitive features	Total alkaloids + protein	Addition feature	Chlorine/potassium	Division feature
Glucose	Primitive features	Total alkaloids − chlorine	Subtraction feature	Total nitrogen/nicotine	Division feature

#### 2.3.2 Key feature selection methods

A three-stage screening process was designed in this study.^21^ The specific calculation methods and parameter settings at each stage were as follows: firstly, to evaluate the discrimination ability of features among different aroma types, the separation index was calculated using the formula *f* = 1.18 × (Δ*X*)/(*W*_1_ + *W*_2_), where Δ*X* represented the distance between the central points of frequency distribution peaks of a certain feature in two aroma types, and *W*_1_ and *W*_2_ referred to the half-peak widths of frequency distribution peaks of the two aroma types respectively; features with *f* > 0.8 were retained.^22^ Secondly, the RandomForestClassifier was constructed with the following parameters:^23^*n*_estimators = 100, min_samples_split = 2, min_samples_leaf = 1, random_state = 42.5-fold stratified cross-validation was adopted for model training and evaluation, and feature importance was calculated based on the contribution of features to aroma classification, with the top 10 important features retained. Finally, Pearson correlation analysis was employed to remove redundant features; for feature pairs with an absolute correlation coefficient exceeding 0.8, one feature was eliminated by combining the random forest importance scores and the substance content levels in the samples. All the above feature screening processes were implemented in the Jupyter environment (Python = 3.9, NumPy = 1.26.4).

#### 2.3.3 Model construction and performance evaluation methods

**Fig. 1:**
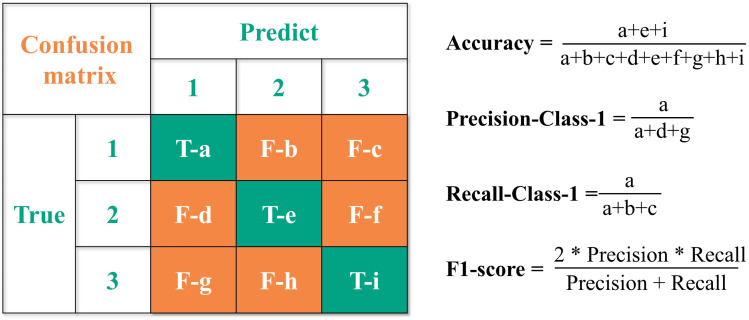
Evaluation principle of confusion matrix.

Six classification models were established on the Jupyter platform (Table 3), namely Logistic Regression (LR), Decision Tree (DT), Random Forest (RF), Extreme Gradient Boosting (XGBoost), *K*-Nearest Neighbor (*K*NN) and Partial Least Squares Discriminant Analysis (PLS-DA). During the data preprocessing stage, feature capping treatment was conducted first, followed by *Z*-score standardization to transform the processed features into dimensionless features with a mean of 0 and a standard deviation of 1, thereby eliminating the interference of feature dimension differences on model performance.^24^ Subsequently, the dataset was divided into a training set and an independent test set in a stratified ratio of 8 : 2 according to aroma types, with the independent test set reserved for the final model performance evaluation. In the model training stage, 5-fold stratified cross-validation was adopted, which ensured that the aroma type distribution proportion in each fold of the dataset was consistent with that in the original dataset, and effectively avoided validation bias caused by single dataset division. The models were evaluated using the confusion matrix, with four metrics adopted as model performance indicators: accuracy, recall, precision, and *F*1-score. The evaluation principle is shown in Fig. 1; lowercase letters in the green diagonal area represent the number of correctly classified samples, while those in the orange area represent the number of incorrectly classified samples.^25^ Given the sample distribution imbalance in the dataset, *F*1-score was utilized as the core evaluation metric. The optimal model for the Sichuan dataset was selected by calculating the mean values of 5-fold cross-validation results, and genetic algorithm (GA) was employed to perform hyperparameter optimization for this optimal model. GA are a class of stochastic search algorithms that simulate the principles of natural selection in the natural world. By mimicking the processes of natural selection, crossover, and mutation, they iteratively search for optimal solutions in high-dimensional parameter spaces. GA offer superior global search capabilities and higher computational efficiency for multi-parameter optimization problems; they can effectively avoid getting stuck in local optima and significantly improve the generalization performance of random forest models.^26^

**Table 3:** Model parameters

Model	Parameters
LR	‘*C*’ = 1.0, class_weight = balanced, random_state = 42
DT	ccp alpha = 0.0, class_weight = balanced, random_state = 42
RF	*n*_estimators = 100, min_samples_leaf' = 1, min_samples_split = 2
XGBoost	*n*_estimators = 100, class_weight = balanced, objective = multi softprob
KNN	*n*_neighbors = 5, algorithm = auto, leaf size = 30
PLS-DA	max_iter = 500, *n*_components = 3, scale = false

#### 2.3.4 Visual analysis of key features

The SHAP value (version 0.44.0) interpretation framework based on game theory was introduced in Jupyter to perform global model interpretation and individualized analysis of key features. For the final selected model, an interpreter is chosen for adaptation; samples from the training set were selected as the background dataset for calculating the marginal contribution of features; the interpreter is used to calculate the SHAP values for each key feature on the test set samples one by one, quantifying the positive or negative contribution of each feature to the model's prediction results, as well as the magnitude of these contributions; based on the calculated SHAP values, global feature importance maps, swarm plots and feature dependency maps are generated to conduct a global interpretation of the model and an individualized analysis of key features.^27,28^ To verify the classification performance of the screened key features, Origin software (version 2026) was utilized to visualize the results of Principal Component Analysis (PCA).

## 3 Results and analysis

### 3.1 Key feature selection

#### 3.1.1 Degree of separation analysis

Degree of separation analysis was conducted on the four datasets generated after feature derivation, with results presented in Table 4. The number of features with *f*_02_ > 0.8 is significantly higher than the number of features with *f*_01_ > 0.8 or *f*_12_ > 0.8 in all four datasets. This suggests that most features exhibit greater discriminative power in distinguishing between fresh-sweet aroma and mellow-sweet aroma than in distinguishing between fresh-sweet aroma and honey-sweet or between honey-sweet and mellow-sweet aromas. Among original features, rutin, chlorogenic acid, anatabine, and serine showed good separation capacity; however, the feature count in the 3 composite datasets remains substantial. Thus, features with *f* > 0.8 were retained for random forest importance analysis.

**Table 4:** Results of feature separability^a^

Dataset	Number of features		
	*f*_01_ > 0.8	*f*_12_ > 0.8	*f*_02_ > 0.8
Primitive features	1	1	4
Additive composite feature	39	39	112
Subtractive composite feature	37	34	113
Division composite feature	18	14	80

a 1. Separability *f* = 1.18 × the difference in center points of adjacent peaks/the sum of the half peak widths of adjacent peaks. 2. *f*_01_ represents the fresh-sweet aroma and the honey-sweet aroma, *f*_12_ represents the honey-sweet aroma and the mellow-sweet aroma, *f*_02_ represents the fresh-sweet aroma and the mellow-sweet aroma. 3. When *f* > 0.8, it indicates that it has a certain separation ability.

#### 3.1.2 Random forest importance analysis

**Fig. 2:**
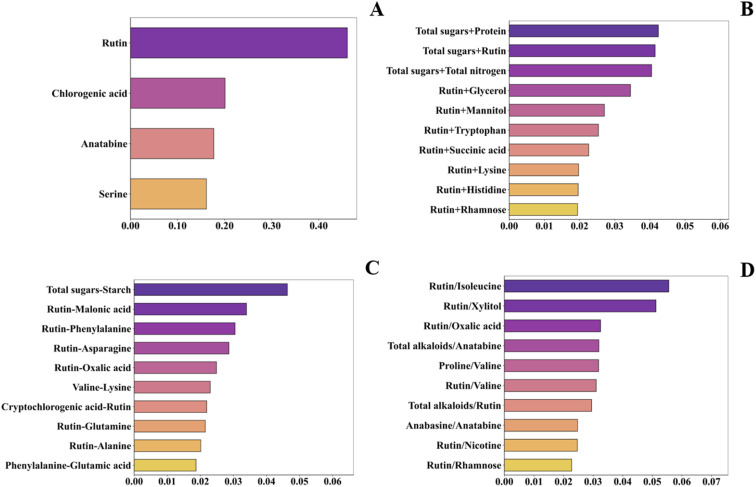
Random forest feature importance plot. (A) Primitive feature importance plot; (B) additive composite feature importance plot; (C) subtractive composite feature importance plot; (D) divisive composite feature importance plot.

Fig. 2 presented feature importance scores based on the RF algorithm for the four datasets after separation analysis. For the primitive feature dataset (Fig. 2A), only rutin, chlorogenic acid, anatabine, and serine were retained. Among additive composite features (Fig. 2B), the top 10 important features were: total sugar + protein, total sugar + rutin, total sugar + total nitrogen, rutin + glycerol, rutin + mannitol, rutin + tryptophan, rutin + succinic acid, rutin + lysine, rutin + histidine and rutin + rhamnose. For subtractive composite features (Fig. 2C), the top 10 important features included: total sugar − starch, rutin − malonic acid, rutin − phenylalanine, rutin − asparagine, rutin − oxalic acid, valine − lysine, cryptochlorogenic acid − rutin, rutin − glutamine, rutin − alanine and phenylalanine − glutamic acid. For divisive composite features (Fig. 2D), the top 10 important features were: rutin/isoleucine, rutin/xylitol, rutin/oxalic acid, total alkaloids/anatabine, proline/valine, rutin/valine, total alkaloids/rutin, anabasine/anatabine, rutin/nicotine and rutin/rhamnose.

#### 3.1.3 Pearson correlation analysis

**Fig. 3:**
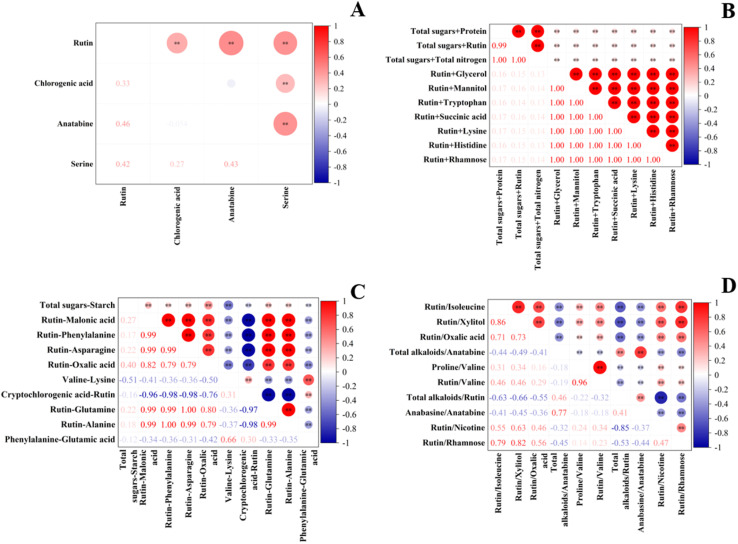
Correlation heatmap. (A) Primitive feature correlation map; (B) additive composite feature correlation map; (C) subtractive composite feature correlation map; (D) divisive composite feature correlation map.

To reduce the interference of multicollinearity in the model, correlation analysis was performed on the feature datasets retained in Section 3.1.2, and the correlation heatmap is shown in Fig. 3. The results indicated that the absolute values of the correlation coefficients among the four original features (rutin, chlorogenic acid, anatabine, serine) were all below 0.8. By contrast, 24, 17 and 3 feature pairs with absolute correlation coefficients greater than 0.8 were observed in the additive, subtractive and divisive composite feature datasets, respectively. Combined with random forest feature importance and substance content levels in the samples (content > 1 mg g^−1^), nine key features were ultimately identified for subsequent modeling: rutin, chlorogenic acid, anatabine, total sugar + protein, rutin − malonic acid, total sugar − starch, rutin/ oxalic acid, total alkaloids/anatabine, and total alkaloids/rutin.

#### 3.1.4 Key feature PCA analysis

**Fig. 4:**
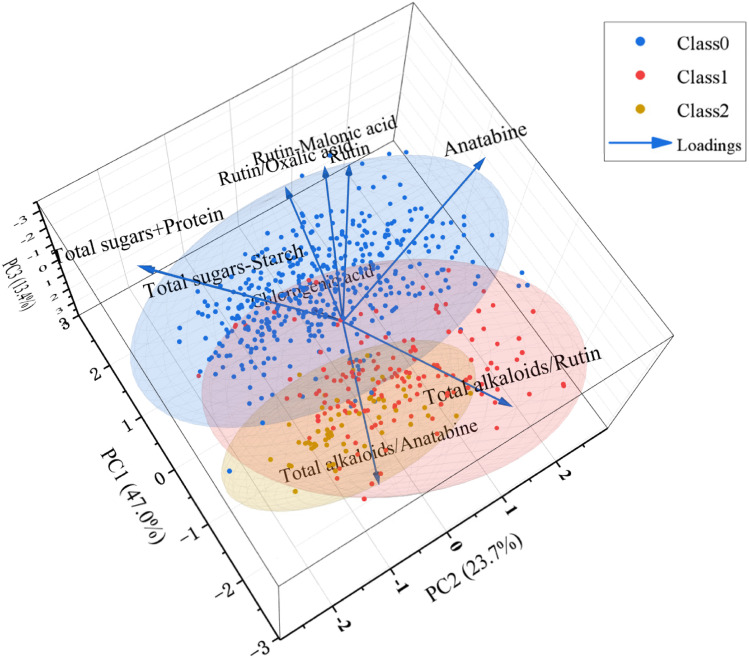
PCA score plot and loading plot of FCT samples across different aroma types.

PCA were performed on the key feature datasets to visually display the distribution patterns of chemical components used for modeling in the three aroma types FCT samples. Fig. 4 presented the PCA biplot integrating the score and loading plots for the three aroma types, which simultaneously presents the spatial distribution of samples and the contribution of each chemical feature to aroma discrimination. Principal Components (PC1, PC2, PC3) explained 47.0%, 23.7% and 13.4% of the total variance, respectively, with a cumulative variance explanation rate of 84.1%, effectively covering most of the original information. The confidence ellipses of the three aroma-type samples showed a certain degree of separation in the three-dimensional space.

However, partial overlap existed among the three groups, indicating that the three aroma type FCTs had both unique properties and shared characteristics. In the loading plot, the length and direction of the loading vectors reflected the magnitude and direction of the influence of each feature on aroma differentiation, respectively. The loading plot indicated that rutin, anatabine, total sugar + protein, rutin − malonic acid, total sugar − starch and rutin/oxalic acid contributed significantly to fresh-sweet aroma type, while chlorogenic acid, total alkaloids/rutin and total alkaloids/anatabine showed remarkable contributions to honey-sweet and mellow-sweet.

### 3.2 Model construction and performance analysis

#### 3.2.1 Performance analysis of different models

Based on the key feature dataset of Sichuan FCT, six machine learning algorithms (KNN, RF, DT, LR, XGBoost, and PLS-DA) were used for model training and performance comparison, with the results listed in Table 5. Taking *F*1-score as the core evaluation metric, DT and PLS-DA showed relatively weak performance, with *F*1-scores of 75.2% and 59.5%, respectively. The *F*1-scores of the other four models all exceeded 80%, among which the RF model was optimal with an *F*1-score of 83.8%. In terms of accuracy, precision and recall, RF performed outstandingly: it ranked first in both accuracy (90.5%) and precision (86.3%), and third in recall (81.8%), indicating that RF could maintain high classification accuracy and discrimination precision under imbalanced sample conditions. XGBoost ranked second in recall (82.1%), but its accuracy and precision were lower than those of RF. KNN ranked third in accuracy, second in precision and fourth in recall. LR achieved the highest recall, while its other indicators were moderate. Considering all indicators comprehensively, the RF model presented the best overall performance on the Sichuan FCT dataset. Therefore, Random Forest (RF) was selected as the final model for aroma classification of Sichuan FCT.

**Table 5:** Evaluation scores of different model metrics

Models	*F*1-score	Accuracy	Precision	Recall
RF	0.838	0.905	0.863	0.818
XGBoost	0.828	0.893	0.837	0.821
KNN	0.822	0.889	0.838	0.808
LR	0.810	0.873	0.787	0.843
DT	0.752	0.846	0.757	0.751
PLS-DA	0.595	0.832	0.711	0.600

#### 3.2.2 Genetic algorithm optimization

To further reduce model overfitting and improve generalization ability, GA was employed for hyperparameter optimization of the RF model. The GA was set with an initial population of 100, a maximum number of iterations of 50, and an error precision of 1 × 10^−6^. Given the large number of adjustable hyperparameters in RF, six key hyperparameters were selected for global optimization based on preliminary experiments and parameter tuning, while the other hyperparameters were set to their default values. The optimization results are listed in Table 6.

**Table 6:** RF hyperparameter value range and GA optimization results

Hyperparameter	Value range	Optimization result
*n*_estimators	(50, 200)	111
max_depth	(3, 20)	10
min_samples_split	(2, 20)	7
min_samples_leaf	(1, 10)	3
max_features	(0.1, 1.0)	0.405
bootstrap	(True/false)	True

#### 3.2.3 Model performance evaluation of GA-RF

The training set was used to verify the learning and fitting capability of the model, while the test set was adopted to evaluate its generalization performance. Considering the sample imbalance in the dataset, the sample size of each aroma type in the test set was extracted in accordance with the classification proportion of the whole dataset. As shown in Table 7, the established GA-RF model achieved a high *F*1-score of 88.3%, and the overall accuracy, precision and recall for the three aroma types were 93.5%, 88.2% and 88.9%, respectively. Compared with the original RF model (*F*1-score: 83.8%; accuracy, precision and recall: 90.5%, 86.3% and 81.8%, respectively), all evaluation indicators of the GA-RF model were significantly improved, showing better overall performance. This optimized model enables high-precision discrimination of the three flue-cured tobacco aroma types using only nine key features, greatly reducing the number of detection indicators.

**Table 7:** Evaluation index scores of GA-RF model

Aroma type	*F*1-score	Accuracy	Precision	Recall
Fresh-sweet aroma	0.975	0.951	1	0.951
Honey-sweet	0.892	0.967	0.828	0.966
Mellow-sweet	0.782	0.750	0.818	0.75
Average	0.883	0.935	0.882	0.889

**Fig. 5:**
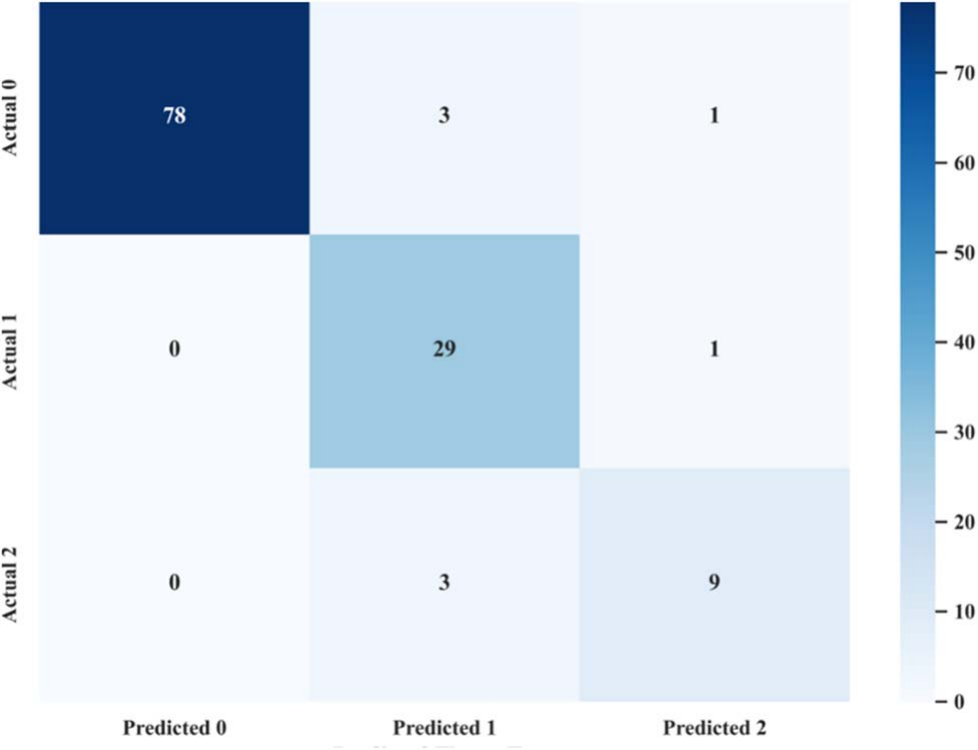
RF – confusion matrix.

The GA-optimized RF model effectively alleviated overfitting and significantly improved generalization ability, with its classification performance summarized in the confusion matrix (Fig. 5). The model's misclassification cases were as follows: for the fresh-sweet aroma, 4 samples were misclassified—3 were misjudged as honey-sweet aroma, and 1 as mellow-sweet aroma; for the honey-sweet aroma, 1 sample was misclassified, which was judged as mellow-sweet aroma; for the mellow-sweet aroma, 3 samples were misclassified, all of which were judged as honey-sweet aroma. This may be attributed to varying degrees of feature overlap between fresh-sweet aroma and honey-sweet, as well as between honey-sweet and mellow-sweet aromas, leading to slight deviations in the model's judgment.

### 3.3 Key feature analysis of SHAP value

#### 3.3.1 Global interpretation analysis

**Fig. 6:**
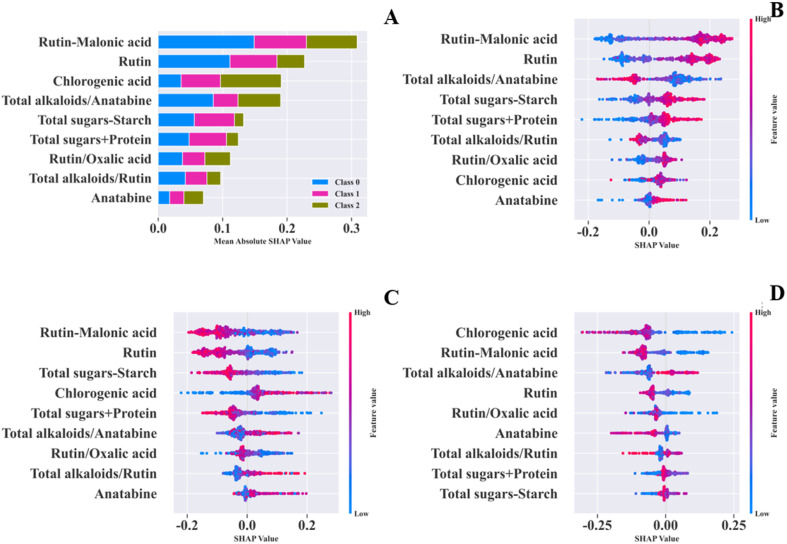
Random forest is based on the global interpretation plot of SHAP value. (A) Summary plot; (B) fresh-sweet aroma bee swarm plot; (C) honey-sweet aroma bee swarm plot; (D) mellow-sweet aroma bee swarm plot.

In addition to evaluation metrics, model transparency and interpretability also serve as supplementary criteria for assessing its reliability. The global feature importance plot (Fig. 6A) quantifies feature importance using mean absolute SHAP values; the bee swarm plots (Fig. 6B–D) illustrate the positive and negative contributions of each feature to different aroma types (colored dots represent the feature content levels of samples, and SHAP values indicate the magnitude and direction of the contributions).^29^

Fig. 6A shows that rutin − malonic acid has the highest mean absolute SHAP value, serving as the core feature for classification in the GA-RF model; the other eight features also have relatively high mean absolute SHAP values and play a key auxiliary role. In Fig. 6B–D, the SHAP values of rutin − malonic acid significantly deviate from 0 in the scatter plots of all three aroma types, exerting a significant impact on classification; in Fig. 6B, the high feature values of rutin − malonic acid and rutin correspond to positive SHAP values, driving the model to classify samples as the fresh-sweet aroma type; in Fig. 6C, their high feature values correspond to negative SHAP values, increasing the probability of classifying samples as the non-honey-sweet aroma type; in Fig. 6D, low feature values of chlorogenic acid correspond to positive SHAP values, prompting the model to classify samples as the mellow-sweet aroma type.

#### 3.3.2 Feature dependency analysis

**Fig. 7:**
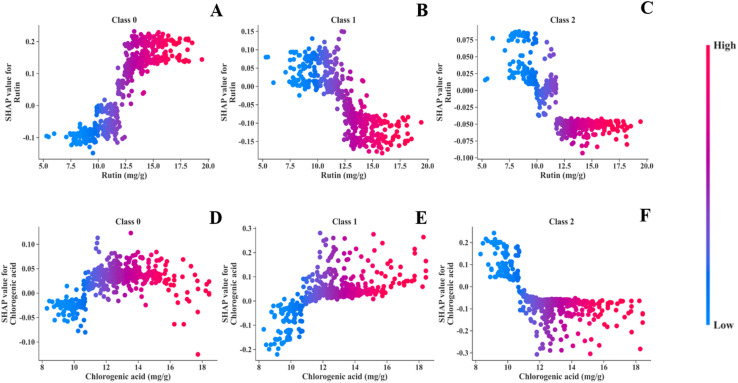
A characteristic dependency diagram of rutin, chlorogenic acid among the three aroma types. (A–C) Is characteristic dependency diagram for rutin, (D–F) is characteristic dependency diagram for chlorogenic acid.

To explore the effects of key features on model classification performance and their value distributions across different aroma types, two original features (rutin and chlorogenic acid) with high contributions to global interpretation and aroma classification were selected for individual feature dependence analysis. Scatter plots were generated for each aroma type (Fig. 7A–C), where the *X*-axis represents the natural value range of the target feature across all samples, and the *Y*-axis denotes the corresponding SHAP values. SHAP values were employed here to quantify the marginal contribution of each feature value to the model's final classification output, enabling direct visualization of how variations in feature values correlate with changes in model prediction outcomes for distinct aroma types. Since tree models are not affected by the dimension differences between features, all indicators were imported as raw data, enabling a more intuitive observation of feature ranges.^30^

Fig. 7A–C are the feature dependence plots of rutin for the three aroma types. The results show that rutin has relatively high SHAP values for its contributions to the fresh-sweet and honey-sweet aromas: its maximum positive contribution to the fresh-sweet aroma reaches 0.2, while it exerts negative contributions to the honey-sweet and mellow-sweet aromas. When the rutin content exceeds 12.5 mg g^−1^, the FCT tends to be of the fresh-sweet type; when the content is below 12.5 mg g^−1^, it is more likely to be of the honey-sweet or mellow-sweet aromas. Fig. 7D–F are the feature dependence plots of chlorogenic acid for the three aroma types. The results indicate that chlorogenic acid has relatively high SHAP values for its contributions to the honey-sweet and mellow-sweet aromas. When the chlorogenic acid content is below 10.5 mg g^−1^, it exerts a positive contribution to the mellow-sweet aroma, while its SHAP values for the fresh-sweet and honey-sweet aromas are negative, and the samples tend to be classified as the mellow-sweet aroma.

Studies have shown that rutin and chlorogenic acid account for a significant proportion of polyphenolic compounds in flue-cured tobacco (FCT), and their contents are closely correlated with ecological conditions and metabolic regulation.^31,32^ In Sichuan, fresh-sweet aroma FCT is mainly produced in the southwestern plateau ecological region, whereas mellow-sweet aroma FCT is primarily grown in the Wuling–Qinba ecological region.^4,18^ The plateau region features intense sunlight, which can effectively promote the synthesis and accumulation of polyphenolic compounds in tobacco.^33^ Moreover, the low-temperature stress caused by sharp temperature drops at night can further induce tobacco to increase polyphenol content through lignification to resist adverse environments.^34^ In contrast to the plateau region, the Wuling–Qinba ecological region presents weaker light intensity, a smaller diurnal temperature difference and insufficient low-temperature stress, which collectively lead to a lower level of polyphenol synthesis and accumulation in tobacco.

## 4 Discussion

In this study, feature derivation was conducted based on 51 original chemical indicators combined with the chemical mechanism of tobacco style formation, expanding the indicators to 3927 features *via* addition, subtraction and division arithmetic operations. Specifically, additive features reflect the synergistic accumulation of aroma precursors, *e.g.*, total sugar + proline, the core substrates of Maillard reaction, jointly regulate the biosynthesis of aroma substances; subtractive features characterize the competitive metabolic pathways, *e.g.*, rutin − malonic acid reveals the trade-off between polyphenols and organic acids sharing synthetic precursors; divisive features indicate the balance of key components, *e.g.*, sugar/nicotine reflects the carbon–nitrogen metabolic balance and smoke acid–base coordination of tobacco leaves. On this basis, a three-tier feature screening framework, namely resolution analysis-RF feature importance ranking-correlation elimination, was established to screen 9 key features, providing a novel paradigm for constructing low-dimensional and high-precision tobacco aroma classification models. Combined with the SHAP interpretable analysis, multi-dimensional visualization clarified the positive/negative contribution patterns and content thresholds of key features for different aromas, offering theoretical support for targeted regulation of tobacco aroma style. In our preliminary study, machine learning was applied to predict tobacco quality grades, yet no aroma classification model was established. Furthermore, the model was constructed solely based on raw chemical indicators and adopted a single feature selection method, failing to thoroughly elucidate the intrinsic association between chemical components and tobacco aroma.^15^ Taking three aroma types (fresh-sweet, honey-sweet, mellow-sweet) of Sichuan FCT as research objects, the GA-optimized RF model was constructed after algorithm comparison and parameter optimization, achieving an aroma classification accuracy of 93.5%, which was significantly superior to previous findings.

Nevertheless, this study has certain limitations. Firstly, the sample distribution is imbalanced with a low proportion of mellow-sweet samples, and feature overlap exists among partial aromas, causing slight classification deviations and relatively low accuracy for mellow-sweet tobacco; future research can expand the sample collection scope to cover more producing areas and improve sample representativeness. Secondly, the current chemical component detection depends on conventional methods with complicated procedures and high costs. In follow-up research, relying on the 9 screened key features combined with near-infrared spectroscopy, portable detection equipment or online identification systems can be developed to realize rapid and low-cost analysis of FCT aroma types, providing technical support for the refined classification of tobacco raw materials.

## 5 Conclusion

The results demonstrated that: (1) nine features closely associated with the three aroma types were ultimately screened out *via* the three-tier screening strategy consisting of separation degree analysis, random forest feature importance ranking and correlation analysis, including rutin, chlorogenic acid, anatabine, total sugar + protein, rutin − malonic acid, total sugar − starch, rutin/oxalic acid, total alkaloid/anatabine and total alkaloid/rutin. (2) The RF model was finally selected for the Sichuan FCT dataset through model comparison, and GA optimization was adopted to further boost the model accuracy, with the *F*1-score reaching 88.3% and the overall classification accuracy hitting 93.5%, which drastically reduced the detection cost while stabilizing the model performance. (3) Visual interpretation based on SHAP values combined with feature dependence analysis further elaborated the impacts of value ranges of key feature indicators on the judgment results of tobacco aroma types. This study provides an efficient approach for the accurate identification of Sichuan FCT aroma types, which is vital for promoting the efficient utilization of tobacco raw materials.

## Author contributions

Zhiying Wang: writing – original draft. Shouchen Yang: investigation. Xiaoting Wang: resources. Jun Qiu: investigation. Jianmin Cao: writing – review & editing, supervision, project administration, formal analysis. Xianwei Hao: funding acquisition.

## Conflicts of interest

The authors declare that they have no known competing financial interests or personal relationships that could have appeared to influence the work reported in this paper.

## Data Availability

The code files for the paper are available on github: https://github.com/JianminCao/Prediction-and-Visual-Analysis-of-Flue-Cured-Tobacco-Aroma-Types-Based-on-Machine-Learning--RSC.git. The raw data files are available here: https://www.scidb.cn/s/iqErYr.
